# Is a sub 7-h Ironman^TM^ possible?

**DOI:** 10.3389/fspor.2022.866599

**Published:** 2022-08-25

**Authors:** Antoine Jolicoeur Desroches, Eric D. B. Goulet

**Affiliations:** ^1^Faculty of physical activity sciences, University of Sherbrooke, Sherbrooke, QC, Canada; ^2^Research Centre on Aging, University of Sherbrooke, Sherbrooke, QC, Canada

**Keywords:** triathon, Ironman™, performance, running, cycling, swimming

## Introduction

An Ironman™ (IM) triathlon is an ultra-endurance event consisting in swimming 3.8, cycling 180 and running 42.2 km. The first ever IM triathlon was held in Hawaii in 1978 and won in a time of 11 h 46 min and 58 sec (Millet et al., 2007; Lepers, 2008). Since then, and especially more so following the first IM event in the continental United States held in Lake Placid in 1999, the IM distance triathlon has grown exponentially in popularity and the best finishing time is significantly faster. To this effect, the men's world record is currently held by the 2008 Olympic champion, Jan Frodeno, who completed the distance in a time of 7 h 27 min and 53 sec, which was achieved in 2021 in Germany in an event specifically designed to break the world record called the *Zwift Tri Battle Royale* (ZTBR). This record was broken on the same year by the 2021 Olympic champion Kristian Blummenfelt in a time of 7 h 21 min and 12 sec at IM Cozumel, but the swim was current-assisted, so it may be argued that this time should not be considered as the world record.^1^On the other hand, the women's world record is held by Chrissie Wellington; she completed the distance in 8 h 18 min and 13 sec.

Just as people wondered if it would be possible to break the 4 min barrier for the mile (Denison, 2006) or the 2-h barrier for the marathon (Joyner et al., 2011; Hoogkamer et al., 2017), people within the triathlon community are starting to wonder whether it would be possible to break the 7-h mark for the IM distance. Projects such as the *Breaking 2* financed by Nike, and the *Ineos 1:59 challenge*, supported by the chemical company Ineos, are events put in place with the goal of creating a hype surrounding an extraordinary sporting feat, which, ultimately, are used by the supporting companies as promotional vehicles and marketing opportunities. Recently, the campaign ≪ *Defy the Impossible* ≫ from which derives the ≪ *Sub 8* ≫ and ≪ *Sub 7* ≫ projects, the latter which represents the main focus of this article, were created with the goal of breaking sometime in 2022 the 7-h barrier for the men and the 8-h barrier for the women for an IM.

We are aware of no scientific writing which attempted to detail from a theoretical point of view whether the breaking of the 7-h mark for the IM is possible. Therefore, the first objective of this article was to elucidate, through the use of predictive analyses, whether an IM distance triathlon under 7-h might be achieved in 2022 without external assistance and with the current swimming, cycling, and running equipment. Our different analyses showed that this is very unlikely, which led to the second goal of this paper that was to demonstrate how external aid should be deployed to achieve the breaking of the 7-h mark. The last part of this article is dedicated to race organizers and expose the key course characteristics and meteorological conditions we believe are required to optimize the chance of success of this event.

## Is a sub 7-h IM possible?

Such to determine whether a sub 7-h IM distance triathlon is achievable without any external help and while using the currently available racing equipment, we used different predictive approaches where we first analyzed the evolution of the fastest times recorded in the history of the IM since 1989 (prior to 1989, archives related to the IM best times are largely missing). Second, we looked at the progression of the racing times registered at the IM event held in Roth from 1990 to 2019, which is recognized as one of the fastest IM race-course in the world. Third, we summed the best swimming, running, and cycling times clocked from all IM races confounded since the inception of the distance. And finally, we predicted the best possible time that could be achieved for the IM distance when doubling the half-IM world record time, while correcting for a computed slowing factor inherent to the doubling of the distance.

### Evolution of the fastest IM times from 1989 to 2021

**Figure 1:**
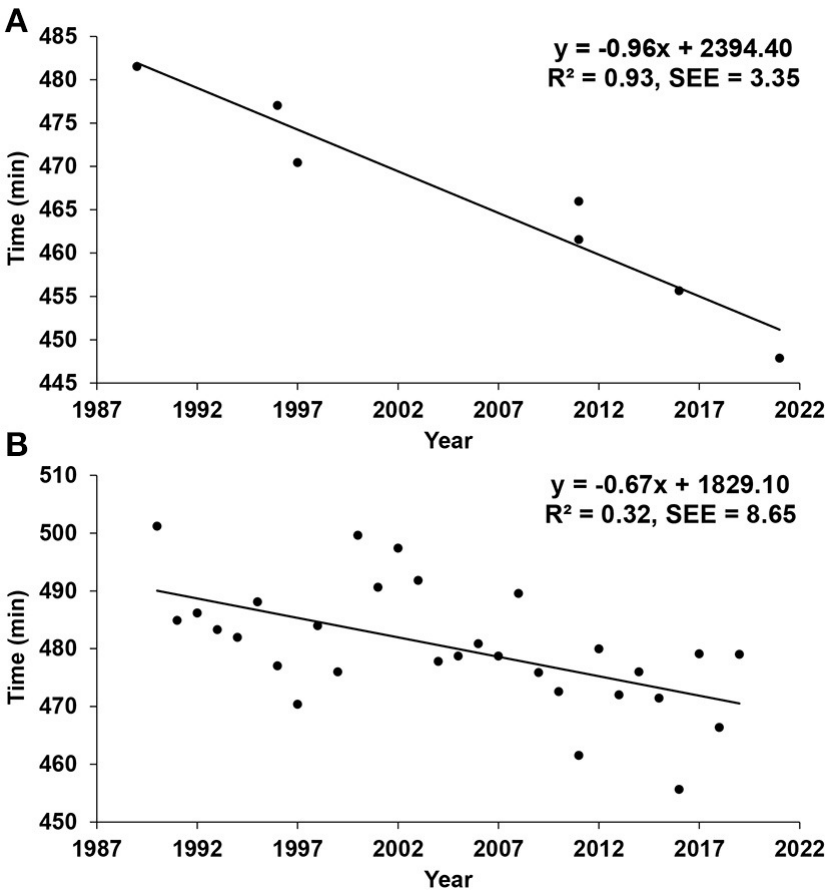
**(A)** Prediction of the best Ironman™ finishing time from the best times achieved over the distance from 1989 to 2021. **(B)** Prediction of the best Ironman™ finishing time from the best times achieved each year over the distance during IM Challenge Roth from 1990 to 2019. SEE, standard error of the estimate.

Table 1 reports the best IM completion times from 1989 to 2021. A time of 7-h would represent an improvement of 6.6% compared to the current world record of 7 h 27 min and 53 sec, which is of considerable importance provided that from 1989 to 2021, the IM world record has only improved by 7.5%, as can be computed from observations made in Table 1. Based on the evolution of the IM world record times over those years, the racing times declined only on average by 1 min and 03 sec per year. Figure 1A illustrates a scatterplot showing the evolution of the IM world record times between 1989 and 2021. The trend of the regression line suggests that there is still room for improving the IM world record time; indeed, over the past 10 years, each new world record has fallen from the previous by an average of ~6 min. Moreover, the time improvement over that time-period has been of ~18 min, compared to ~16 min over the previous 20 years. Interestingly, the regression line predicts that in 2022 the fastest IM time is expected to be between 7 h 39 min and 59 sec and 7 h 26 min and 35 sec, the latter which is 0.3 % faster than the current world record, which was achieved during an event specifically designed to break the previous world record of 7 h 35 min and 39 sec. However, this time is still significantly slower than a sub 7-h IM. Importantly, based on the calculated trend from the best IM finishing times observed over the past 32 years, a sub 7-h IM completed without external assistance could theoretically not be achieved at least before the year 2049.

**Table 1:** World record Ironman™ triathlon performances.

**Year**	**Athletes**	**Location**	**Total time (h:min:sec)**
1989	Dave Scott	Lake Biwa, Japan	8:01:32
1996	Lothar Leder	Roth, Germany	7:57:02
1997	Luc Van Lierde	Roth, Germany	7:50:27
2011	Marino Vanhoenacker	Klagenfurt, Austria	7:45:58
2011	Andreas Raelert	Roth, Germany	7:41:33
2016	Jan Frodeno	Roth, Germany	7:35:39
2021	Jan Frodeno	Algäu, Germany	7:27:53
2021	Kristian Blummenfelt	Cozumel, Mexico	7:21:42*

*For the purpose of this article, the time of 7 h 21 min and 42 sec achieved by Kristian Blummenfelt is not considered the world record since the swim was current-assisted.

### IM Challenge Roth: Racing time progression between 1990 and 2019

IM Challenge Roth has produced the most IM world records since 1990; therefore, we believe that these numbers represent a good source of information for estimating when a sub 7-h IM may be accomplished. Table 2 demonstrates the winners' completion times for IM Challenge Roth from 1990 to 2019.^2^ The slowest and fastest times achieved over this timespan were respectively 8 h 21 min and 13 sec and 7 h 35 min and 39 sec. Hence, an improvement in time of 10%, or 1 min 30 sec per year was observed over this period of 30 years, which fits nicely well with the results observed regarding the improvement in IM world record times between 1989 and 2021. As shown in Figure 1B, a regression line built from the winning times over the past 30 years at IM Challenge Roth demonstrates that the breaking of the 7-h barrier for IM Roth is unlikely to occur at least prior to 2077, based on an associated measurement time error of ±17 min and 17 sec.

**Table 2:** Evolution of the racing time of the winners of the Challenge Roth Ironman™ triathlon from 1990 to 2019.

**Years**	**Total time**	**Years**	**Total time**	**Years**	**Total time**
**1990–1999**	**(h:min:sec)**	**2000–2009**	**(h:min:sec)**	**2010–2019**	**(h:min:sec)**
1990	8:21:13	2000	8:19:38	2010	7:52:36
1991	8:04:54	2001	8:10:39	2011	7:41:33
1992	8:06:12	2002	8:17:25	2012	7:59:59
1993	8:03:19	2003	8:11:50	2013	7:52:01
1994	8:01:59	2004	7:57:50	2014	7:56:00
1995	8:08:07	2005	7:58:45	2015	7:51:28
1996	7:57:02	2006	8:00:52	2016	7:35:39
1997	7:50:24	2007	7:58:45	2017	7:59:07
1998	8:03:59	2008	8:09:34	2018	7:46:23
1999	7:56:00	2009	7:55:53	2019	7:59:02

### Summation of the world's fastest IM swimming, running, and cycling times

Another reasonable way to estimate whether a sub 7-h IM is possible without external assistance is to combine and sum the world's fastest IM swimming, cycling, and running times ever achieved by any given athletes in any given IM races. Because these times were presumably achieved by discipline specialists and potentially under the best conditions possible, it is very unlikely that even with the best preparation possible a single IM triathlete could align and match or even best each of those times.

The fastest, non-current-assisted swimming time in an IM triathlon was achieved with a wetsuit, in a lake, by Jan Sibbersen, in a time of 42 min and 17 sec in 2004. The fastest official cycling time in an IM, achieved at the ZTBR in 2021 by Jan Frodeno, is 3 h 55 min and 22 sec.^3^ Finally, Gustav Iden completed the fastest official IM marathon time (2 h 34 min and 50 sec) in 2021 at IM Florida.^4^ The summation of these times, i.e., 7 h 12 min and 29 sec, reveals the fact that even the best times achieved over time within each of the disciplines that make up triathlon would not be sufficient to break the 7-h mark. And one must take into account that this overall time does not include the transition times.

### Prediction of IM time from the half-IM world record time

The current half-IM world record is held by Kristian Blummenfelt. The Norwegian athlete completed the 2018 Bahrain half-IM in a time of 3 h 29 min and 04 sec. This figure highlights the fact that an athlete looking to break the 7-h mark would need to sustain that pace for twice the distance, which would represent an extraordinary accomplishment. Indeed, by doubling the swimming, cycling, and running times a total racing time of 6 h 54 min and 38 sec is obtained.^5^ However, as shown in Table 3, when the fastest IM and half-IM times of the current top 20 triathletes in the world competing in both IM and half-IM are compared, it can be observed that, on average, the IM time is 7.3 ± 4.2% slower than the doubling of the half-IM time.^6^ Based on this slowing factor, our calculations indicate that Blummenfelt's predicted IM time would be 7 h 24 min and 58 sec, with a margin of error of ±18 min and 38 sec. Blummenfelt completed his first IM in 2021 (with a current-assisted swim) with a time of 7 h 21 min and 12 sec. Therefore, in order to break the 7-h barrier, he would need to be 5.9% faster than his predicted time, which is very unlikely considering, as reported above, that the IM world record has only improved by 7.5% over the past 32 years.

**Table 3:** Comparison of the fastest half-Ironman™ and Ironman™ triathlons of some of the best current long-distance triathletes in the world.

**Athletes**	**Half-IM best times (h:min:sec)**	Doubling of the Half-IM best times (h:min:sec)*	**IM best times (h:min:sec)**	**Difference (%)^†^**
Andreas Dreitz	3:40:12	7:16:54	7:53:06	7.7
Bart Aernouts	3:44:38	7:25:46	7:55:12	6.2
Braden Currie	3:23:33	6:43:36	7:54:58	15
Daneil Baekkegard	3:29:18	6:55:06	7:52:58	12.2
Denis Chevrot	3:41:14	7:18:58	7:51:00	6.8
Florian Angert	3:41:27	7:19:24	7:45:05	5.5
Gustav Iden	3:29:25	6:55:20	7:42:56	10.3
Jackson Laundry	3:39:50	7:16:10	8:26:00	13.8
Jan Frodeno	3:36:31	7:09:32	7:27:53	4.1
Jan Van Berkel	3:40:49	7:18:08	7:39:40	4.7
Joe Skipper	3:55:32	7:47:34	7:53:52	1.3
Kristian Hogenhaug	3:50:37	7:37:44	7:37:46	0
Kyle Smith	3:39:43	7:15:56	8:08:53	10.8
Leon Chevalier	3:50:20	7:37:10	7:57:02	4.2
Lionel Sanders	3:38:18	7:13:06	7:43:30	6.6
Matt Hanson	3:46:48	7:30:06	7:39:25	2
Patrick Lange	3:43:46	7:24:02	7:45:21	4.6
Rasmus Svenningsson	3:29:18	6:55:06	7:51:33	12
Sam Appleton	3:43:58	7:24:26	8:09:54	9.3
Sam Long	3:37:34	7:11:38	7:55:33	9.2
**Average**	**3:40:09**	**7:16:47**	**7:51:35**	**7.3** **±4.2**^**&**^

*Half-IM time × 2, substracting a time of 3 min and 30 sec for the transition times.^†^IM best time–doubling of the half-IM best time.

^&^Average ± standard deviation.

Based on the computations presented above, we can therefore conclude with relative confidence that a sub 7-h IM is very unlikely to be achieved without external assistance anytime soon. But a question remains; is it even reasonable to believe that an IM under 7-h could be completed with external help. The next part of the article will focus on this given question.

## Can a sub 7-h IM be achieved with assistance?

A prerequisite to the determination that an IM could indeed be achieved in or under a time of 7-h is to first correctly partition the amount of time that should be attributed to each of the single parts of the race, that is the swimming, cycling, running and transition times. We underwent this exercise by contrasting the finishing times for each of the three disciplines in relation to the total finishing times of the winner of the IM Challenge Roth triathlon from 1990 to 2019. As demonstrated in Table 4, we determined that the swimming, cycling, and running times represent, on average, 10.2, 54.9, and 34.9% of the total completion time of an IM, with a standard deviation for each of the three disciplines ≤ 1%, which underlines the robustness of these observations. From those calculations, a single triathlete performing a 7-h IM would likely achieve times in the vicinity of 42 min and 32 sec for swimming, 3 h 49 min and 32 sec for cycling and 2 h 25 min and 47 sec for running, and the total transition times would need to be <2 min. It immediately jumps to the eyes that this triathlete would require to produce world-record swimming, cycling, and running performances to achieve a sub 7-h IM; this is unlikely to occur in 2022, nor in the near future as well. But those theoretical numbers do expose one important factor in that a sub 7-h IM could only be accomplished with outside help and if substantial time deficits are made during the cycling portion of the race. Indeed, although it is reasonable to believe that a strong swimmer could meet the targeted swimming time of ~42-43 min, it would be utopic to consider that a sub 2 h and 26 min marathon time is achievable.

**Table 4:** Time for each discipline of the winners of the Ironman™ Challenge Roth triathlon from 1990 to 2019 in relation to total racing time.

**Years**	**Total time**	**Swim**	**% Swim**	**Cycle**	**% Cycle**	**Run**	**% Run**
	**(h:min:sec)**	**(h:min:sec)**		**(h:min:sec)**		**(h:min:sec)**	
1990	8:21:13	0:49:14	9.82	4:43:30	56.56	2:48:29	33.61
1991	8:04:54	0:49:38	10.24	4:30:26	55.77	2:44:50	33.99
1992	8:06:12	0:50:52	10.46	4:29:38	55.46	2:45:24	34.02
1993	8:03:19	0:53:23	11.04	4:21:04	54.02	2:48:51	34.94
1994	8:01:59	0:49:41	10.31	4:23:53	54.75	2:48:25	34.94
1995	8:08:07	0:51:11	10.49	4:20:28	53.36	2:56:28	36.15
1996	7:57:02	0:49:33	10.39	4:24:06	55.36	2:43:23	34.25
1997	7:50:24	0:44:51	9.53	4:28:47	57.14	2:36:49	33.34
1998	8:03:59	0:48:16	9.97	4:23:02	54.35	2:52:41	35.68
1999	7:56:00	0:50:59	10.71	4:15:47	53.74	2:49:13	35.55
2000	8:19:38	0:47:43	9.55	4:31:18	54.3	3:00:37	36.15
2001	8:10:39	0:46:51	9.55	4:33:09	55.67	2:50:37	34.77
2002	8:17:25	0:50:50	10.22	4:32:09	54.71	2:54:26	35.07
2003	8:11:50	0:50:42	10.31	4:28:57	54.68	2:52:08	35
2004	7:57:50	0:47:59	10.04	4:28:27	56.18	2:41:22	33.77
2005	7:58:45	0:47:33	9.93	4:24:32	55.25	2:46:38	34.81
2006	8:00:52	0:46:53	9.75	4:27:51	55.7	2:46:06	34.54
2007	7:58:45	0:49:45	10.39	4:17:58	53.89	2:46:39	34.81
2008	8:09:34	0:48:47	9.96	4:31:59	55.55	2:48:49	34.48
2009	7:55:53	0:50:30	10.61	4:22:56	55.25	2:42:30	34.15
2010	7:52:36	0:46:51	9.91	4:24:48	56.03	2:40:53	34.04
2011	7:41:33	0:46:18	10.03	4:13:11	54.85	2:42:06	35.12
2012	7:59:59	0:47:41	9.93	4:31:07	56.49	2:41:13	33.59
2013	7:52:01	0:46:05	9.76	4:16:24	54.32	2:49:35	35.93
2014	7:56:00	0:48:58	10.29	4:21:23	54.91	2:45:40	34.8
2015	7:51:28	0:47:33	10.09	4:10:55	53.22	2:53:02	36.7
2016	7:35:39	0:45:22	9.96	4:09:25	54.74	2:40:36	35.25
2017	7:59:07	0:52:55	11.05	4:20:42	54.41	2:45:28	34.54
2018	7:46:23	0:47:59	10.29	4:09:30	53.5	2:48:57	36.23
2019	7:59:02	0:51:28	10.74	4:14:59	53.23	2:52:38	36.04
**Average** **±SD (%)**			**10.2** **±0.4**		**54.9** **±1.0**		**34.9** **±0.9**

SD, standard deviation.

**Figure 2:**
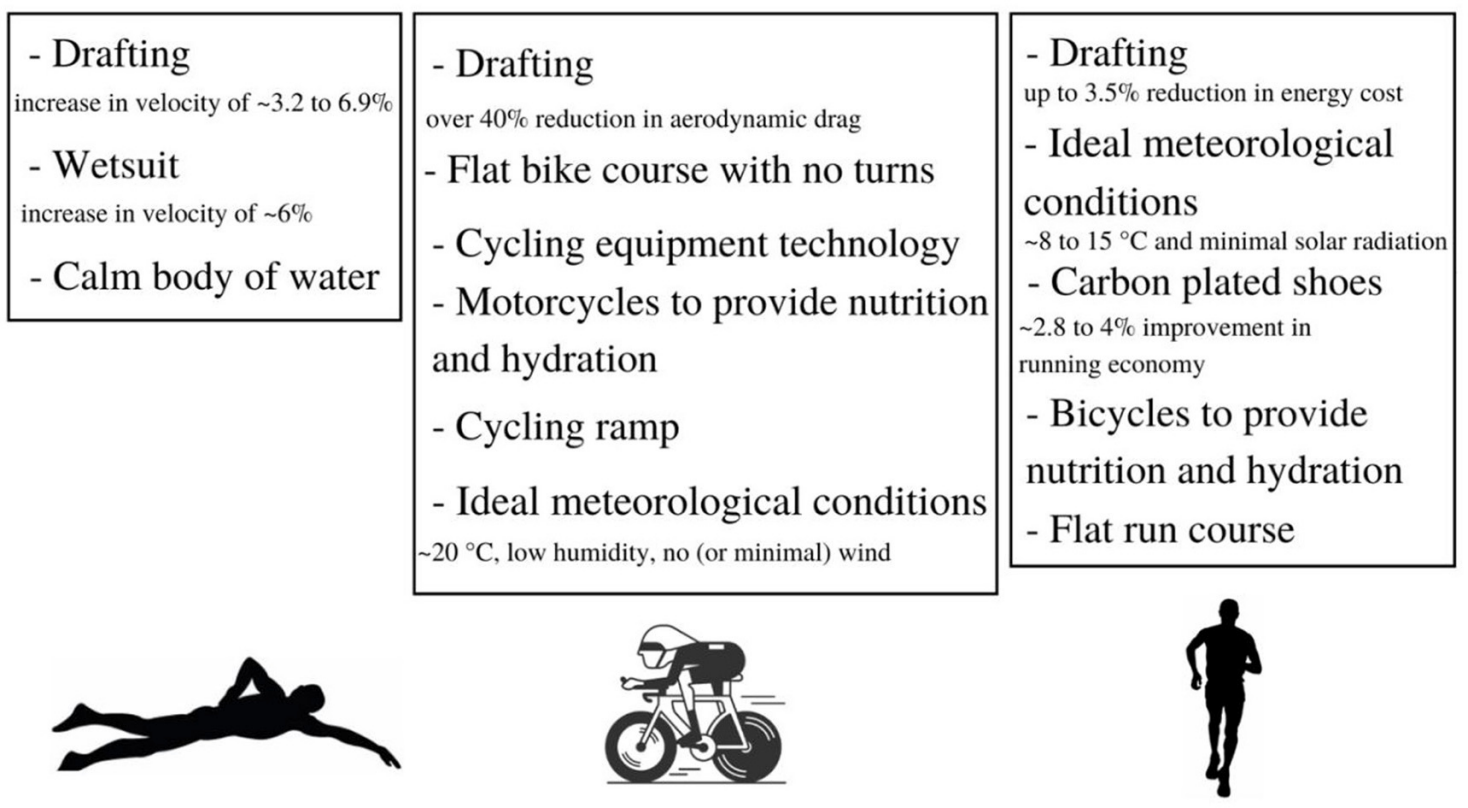
Summary of the different variables that can make a sub-7 Ironman™ performance possible.

In the next sections, an attempt will be made to demonstrate that with external assistance mostly through drafting during the swimming, cycling, and running parts of the race to overcome water or air resistance, a completion time of 7-h for the IM is possible. The different variables that can help make a sub 7-h IM performance possible are summarized in Figure 2. However, this IM performance would not be considered an official IM record according to the World Triathlon Corporation rules. Similarly, the sub 2-h marathon achieved by Eliud Kipchoge is not considered eligible as an official world record. In the current paper, ≪ assistance ≫ is defined as any means that an athlete could be using to maximize its swimming, running and cycling velocity, excluding, however, all forms of help coming from water current, a significant negative elevation for the cycling and the run, magnets or equipment running on or requiring a source of energy from the sun, electricity, gazes or batteries. The term ≪ racing triathlete ≫ is used to refer to the triathlete identified to attempt the breaking of the 7-h mark.

### Swimming

Table 5 shows the best swimming times realized in an IM for each of the years between 2005 and 2021. As mentioned previously, Jan Sibbersen owns the fastest swimming time (Trirating, 2022). What is remarkable is that his record has not been beaten since. It is worth highlighting the fact that this triathlete has been a national level swimmer for 10 years and has achieved personal best times in a 50-meter pool of 1 min and 52 sec for the 200 m, 3 min and 56 sec for the 400 m and 15 min and 47 sec for the 1500 m (Sailfish, n.d.). This may explain why this record still stands. Nevertheless, in 2010 and 2013, wetsuit-assisted swimming times close (<43 min) to that of Sibbersen have been realized by swimmers with no particular competitive background in swimming, at least to our knowledge. Taken together, these information show that without the wearing of a wetsuit, an athlete attempting to break the 7-h barrier is very unlikely to achieve the targeted time of 42 min and 32 sec. Moreover, it seems that an IM swimming time <43 min represents an exception confirming the rule, i.e., that it is tremendously difficult to realize. However, as will be demonstrated below, a strong swimmer capable of achieving a lake- or ocean-based, wetsuit-assisted swimming time of 46-47 min for the 3.8 km distance could potentially achieve this targeted time with the appropriate external help.

**Table 5:** Yearly fastest swimming times in an Ironman™ between 2005 to 2021.

**Year**	**Athletes**	**Event**	**Time (min:sec)**
2005	Jan Sibbersen	IM Austria	44:14
2006	Gilles Reboul	IM France	44:18
2007 2008	Bryan Rhodes Andi Boecherer	IM United Kingdom IM Germany	44:39 43:55
2009	John Flanagan	IM Louisville	44:54
2010	Luke McKenzie	IM Brasil	42:26
2011	Clayton Fettell	IM Cairns	43:48
2012	Luiz Francisco Ferreira	IM Brasil	44:04
2013 2014 2015 2016	Bart Colpaert Luiz Francisco Ferreira Dylan McNeice Dylan McNeice	IM Austria IM Austria IM New Zealand Challenge Wanaka	42:54 44:17 44:26 43:30
2017 2018 2019	Luiz Francisco Ferreira Jesper Svensson Thomas Davis	IM Brasil IM Brasil Challenge Anhui	44:12 43:47 43:48
2020	Josh Amberger	IM Cairns	45:41
2021	Josh Amberger	IM Cairns	43:28

#### Energy cost of swimming

Swimming performance is optimized when propelling cost is maximally reduced. From a pure mathematical standpoint, the energy cost of swimming is calculated by dividing oxygen consumption at a steady state by the corresponding velocity (Zamparo et al., 2005). The energy cost of swimming depends on propelling efficiency, which represents the amount of work necessary to overcome hydrodynamic resistance in relation to the total work required to cover a given distance. Increasing swimming velocity will increase the work necessary to overcome hydrodynamic resistance as the latter increases with the square of the velocity. In addition to the improvement of swimming technique, the wearing of a wetsuit (de Lucas et al., 2000; Zamparo et al., 2005; Tomikawa et al., 2008; Peeling and Landers, 2009) and drafting lead swimmers (Bassett et al., 1991; Chatard et al., 1998; Brisswalter and Hausswirth, 2008) have been shown to reduce the work to overcome hydrodynamic resistance and therefore reduce the energy cost of swimming.

#### Wetsuit

Wearing a wetsuit allows the swimmer to be more horizontal and higher relative to the water surface which, in turn, will act to reduce hydrodynamic resistance (de Lucas et al., 2000; Peeling and Landers, 2009) and the required energy to complete a swimming distance. However, the extent of these effects is dependent upon intrinsic factors related to swimmers such as the swimming technique, propelling efficiency and buoyancy (Chatard et al., 1995). Wearing a wetsuit has been demonstrated to reduce the cost of swimming at 80% of maximal oxygen consumption $\dot{V}$O_2max_) by ~7.5% (Tomikawa et al., 2008) and to increase velocity by ~6% (Gay et al., 2020).

#### Drafting

A lead swimmer creates a depression in the water behind him, which generates a low-pressure gradient. Therefore, the swimmer drafting a lead swimmer encounters lower resistive body drag (Hausswirth and Brisswalter, 2008). The reduction in drag is estimated to vary between ~10 to 26% (Bentley et al., 2007). Many studies have observed lower lactate levels, ratings of perceived exertion and oxygen consumptions when drafting compared to swimming at the same velocity without drafting (Bassett et al., 1991; Chatard et al., 1998). As a result, drafting has been demonstrated to reduce the metabolic cost of swimming by ~5 to 10% and increase velocity by ~3.2 to 6.9% (Bentley et al., 2007). Importantly, the energy saved by drafting a lead swimmer can also have a positive impact on the bike and run portions of the race. Indeed, Delextrat et al. (2003) have observed a 4.8% increase in cycling efficiency after swimming 750 meters at competition pace while drafting a swimmer, compared to without drafting.

Taken altogether, these observations unequivocally illustrate that an acceptable swimming time for an athlete attempting to break the 7-h mark can only be achieved while wearing a wetsuit and using lead swimmers. Considering that the use of drafting could improve swimming performance by an upper limit of 6.9%, then an accomplished swimmer with a demonstrated capacity to achieve a wetsuit-assisted swimming time of 46-47 min during an IM should expect to achieve a swimming time in the vicinity of 42 min and 50 sec to 43 min 45 sec with proper drafting. A swimming time slightly over these figures should not be problematic as important time gains are possible on the bike.

### Cycling

Cycling is the discipline that is the longest, both in distance and in time in an IM. According to Sousa et al. (2019), it is the part of the race that better predicts the total time to complete the distance (R^2^ = 0.69; *p* < 0.001), followed by running (R^2^ = 0.52; *p* < 0.001) and swimming (R^2^ = 0.23; *p* < 0.001). As reported above, our calculation indicates that in order to break the 7-h barrier a triathlete would need to complete the 180 km cycling distance in a time faster than 3 h 49 min and 32 sec such to compensate for the possible time lost accrued during the swimming and running portion of the race vs. the theoretical times. Therefore, the racing triathlete would need to cycle at a velocity > 47.1 km·h^−1^. In comparison, the fastest official time in an IM is 3 h 55 min and 22 sec (45.9 km·h^−1^). This would therefore represent an improvement in cycling time of over 2.5% compared to the actual fastest cycling time achieved in an IM. Table 6 demonstrates the improvement in cycling world record times in an IM between 2005 and 2021. Impressively, between those years, the cycling time fell by 10%. This is likely due to new improvements in technology. Interestingly, the actual record improved over the previous one by 2.4%, which is impressive. However, the use of motorcycles to provide nutrition and hydration, and of a cycling ramp, similar to a velodrome ramp, to allow the triathletes to do a 180° turn without having to slow down, may have been the key contributing factors for the achievement of this fast cycling time. We believe that shaving another 2.5% (or 5 min and 53 sec) off the actual world record may be difficult to reach in 2022 without special accommodations or exceptional race circumstances.

**Table 6:** World record cycling times in an Ironman™ since 2005.

**Year**	**Athletes**	**Event**	**Type of bike course**	**Time (h:min:sec)**
2005	Torbjorn Sindballe	IM World Championships Hawaii	Flat roads with rolling hills	4:21:36
2006	Mitchell Anderson	IM Western Australia	Flat roads	4:18:07
2007	Thomas Hellriegel	Challenge Roth	Flat roads with rolling hills	4:16:18
2009	Normann Stadler	Challenge Roth	Flat roads with rolling hills	4:14:42
2010	Sebastian Kienle	Challenge Roth	Flat roads with rolling hills	4:14:07
2011	Andreas Raelert	Challenge Roth	Flat roads with rolling hills	4:11:43
2012	Andrew Starykowicz	IM Florida	Flat highway	4:04:39
2013	Andrew Starykowicz	IM Florida	Flat highway	4:02:17
2017	Andrew Starykowicz	IM Texas	Flat highway	4:01:14
2021	Jan Frodeno	Zwift Tri Battle Royale	Flat roads	3:55:22

There are three main resistive forces encountered when cycling that influence the power that a cyclist needs to generate to move forward: (1) rolling resistance, (2) gravity and, (3) air resistance (Martin et al., 1998; Crouch et al., 2017). Reducing to a minimum the impact of these forces on the bike and cyclist is the key for the achievement of fast cycling times. The next sections provide cues on how to minimize the effect of resistive forces while cycling and demonstrate how the use of drafting would permit to perform the 180 km portion of the IM at the velocity required to race a sub 7-h IM.

#### Rolling resistance and gravity

The wheels are in contact with the road during cycling, which creates resistance. The rolling resistance is affected by the tire pressure and the vertical load applied on the tire. The vertical load is impacted in proportion to the weight of the cyclist and bicycle (Grappe et al., 1999). However, by optimizing the choice of tire and tire pressure and by choosing a bike course with excellent road surface conditions, it is possible to limit the rolling resistance to a minimum. Of course, the ideal race-course should include minimal elevation gains as the force required to move a bike uphill against the force of gravity is substantially more than that required to ≪ fight ≫ rolling resistance or air resistance. On a flat bike course with an ideal road surface condition, the cyclist weight as well as the bicycle weight will have a minimal impact on performance.

#### Air resistance

The aerodynamic drag (the force applied by the air on an object to resist its motion) depends on the drag coefficient (the efficiency with which an object passes through the surrounding air) and frontal area of the cyclist, the air density and the relative wind speed (Griffith et al., 2014; Crouch et al., 2017). Since aerodynamic drag increases with the square of relative wind speed, as a cyclist goes faster, air resistance becomes a greater factor affecting his rate of forward progression (Faria et al., 2005). At a velocity of 30 km·h^−1^, air resistance represents about 80% of the resistive force encountered by a cyclist (di Prampero, 2000), and up to 90% of the resistive force at higher velocities (Grappe et al., 1997; Griffith et al., 2014).

The drag area represents the combination effect (product) of the drag coefficient and frontal area (Barry et al., 2015a). The drag coefficient depends on body geometry and it is lower for streamlined objects such as an airfoil or a car (Barry et al., 2015b). A cyclist should try to reduce its frontal area and streamline his geometry without compromising too much comfort and the ability to generate power. Indeed, about 80% of the aerodynamic drag of a cyclist on a bicycle is due to the cyclist and the remainder is related to the bicycle, the wheels and the accessories such as the water bottle position on the bicycle (Crouch et al., 2017). Small changes to a cycling position, especially to the placement and angle of the handlebars, can reduce the drag area such to confer a 60-sec improvement over a 40 km time trial, which would translate to a 4 min and 30 sec improvement over 180 km (Jeukendrup and Martin, 2001).

Triathletes use bicycles designed to reduce aerodynamic drag. Indeed, they have an aerodynamic frame, handlebars designed for time trialing and lenticular wheels, which, in total, can save about 60 W of power output at a velocity of 50 km·h^−1^ (Jeukendrup and Martin, 2001). Triathletes competing in IM triathlons do not need to comply with the International Cycling Union (UCI) rules regarding the cyclist position and the cycling equipment (García-López et al., 2008). Therefore, they can optimize their position to reduce their aerodynamic drag as much as possible.

#### Drafting

Drafting behind another cyclist can significantly reduce air resistance (Blocken et al., 2013), but it is not allowed in IM triathlons. However, if external assistance is used for the *sub 7 project*, a group of cyclists could ride in front of the racing triathlete which would allow him to ride at a significantly higher speed and reduce energy expenditure, oxygen consumption (Lukes et al., 2005), heart rate, lactate concentration and perceived exertion, which would also allow the racing triathlete to run faster after cycling (Hausswirth et al., 2001).

The magnitude of the effect of drafting may depend on many factors, such as the number of cyclists in the group, the position of the cyclists, the distance between each cyclist as well as the drag area of the lead cyclists (Lukes et al., 2005). A cyclist drafting as closely as possible behind a lead cyclist may experience a drag reduction of as much as 15-50%, which may reduce to 10-30% at a distance of one bicycle (Crouch et al., 2017). Barry et al. (2015a) studied the aerodynamic drag of 4 cyclists riding in a time-trial position in a team pursuit position. The four riders experienced, respectively, an average drag saving of 5, 45, 55, and 57% at a speed of 65 km·h^−1^. Therefore, a time significantly faster than the theoretical time previously calculated (time of 3 h 49 min and 32 sec) could be achieved.

By taking into consideration body weight, equipment weight, coefficient of drag (CDA), air density (Rho), rolling resistance and slope, we estimated the power output required to complete the cycling portion of the race in different times (between 4 h 00 and 3 h and 40 min) (Table 7) (Martin et al., 1998). For the purpose of illustration, the calculations were done using a body mass of 74 kg and 2 estimated drag coefficients. Based on these numbers, and assuming a conservative reduction in power output of 35-40% due to drafting, the racing triathlete would only need to maintain a power output of 240-268 W to complete the 180 km in 3 h and 40 min, which would be significantly less than the power output required if he were to ride without drafting estimated to be between 370 and 390 W. In turn, this highlights the fact that the alternating lead cyclists would have to be capable of sustaining that amount of power. Hence, it must be borne in mind that, ultimately, the rate of progression of the racing triathlete will be dictated by the quality of the lead cyclists. Indeed, in order to be able to push a power output of 370–390 W for a few minutes when leading the group, the lead cyclists would need to have a critical power (CP) higher than 390 W, which can be expected of professional cyclists (World Tour of professional-continental cyclists) (Bartram et al., 2018).

**Table 7:** Modulation of the cycling time and power output based on different drag coefficient scenarios and on different power reductions from drafting.

**Variables**	**Scenario 1**	**Scenario 2**
Cyclist weight (kg)	74	74
Equipment (bicycle, wheels, etc.) weight (kg)	8	8
Total weight (kg)	82	82
Frontal area (m^2^)	0.3	0.32
Drag coefficient (dimensionless)	0.7	0.7
Drag coefficient x frontal area (CdA)	0.21	0.224
Air density (Rho) (kg/m^3^)	1.226	1.226
Rolling resistance (dimensionless)	0.004	0.004
Slope G (%)	0	0
**Cycling time (h:min:sec)**	**Power output (W)**	
4:00:00	292	308
3:55:00	309	327
3:50:00	328	347
3:49:32 3:40:00	330 370	349 390
3:40:00 with drafting (assuming 35 % power reduction)	256	268
3:40:00 with drafting (assuming 40 % power reduction)	240	251

### Running

In 1997, Luc Van Lierde achieved an outstanding IM world record marathon time of 2 h 36 min and 49 sec. So special was this record that it stood for 22 years until it was beaten in 2019 by a scarce 40 sec. Since then, the world record has improved by another 1 min and 19 sec. Table 8 demonstrates the fastest IM marathon times reached for each of the years between 2005 and 2021. What it clearly illustrates is that running an IM marathon <2 h and 35 min represents and extraordinary accomplishment that has only been realized on one occasion since the inception of this distance.

**Table 8:** Yearly fastest Ironman™ running times between 2005 and 2021.

**Year**	**Athletes**	**Event**	**Time (h:min:sec)**
2005	Gerrit Schellens	IM Lanzarotte	2:44:29
2006	Gerrit Schellens	IM Switzerland	2:43:45
2007 2008	Chris McCormack Timo Bracht	IM World Championship Hawaii IM Germany	2:42:02 2:42:33
2009	Michael Goehner	Challenge Roth	2:41:17
2010	Rasmus Henning	Challenge Roth	2:39:43
2011	Mads Vittrup	IM Copenhagen	2:38:58
2012	Craig Alexander	IM Melbourne	2:38:46
2013 2014 2015 2016	Bart Aernouts Jeff Symonds Victor Del Corral Joe Skipper	IM France IM Canada IM France Challenge Roth	2:37:01 2:40:34 2:42:04 2:38:52
2017 2018 2019	Patrick Lange Jan Frodeno Ben Hoffman	IM World Championship Hawaii IM Germany IM Florida	2:39:59 2:39:06 2:36:09*
2020	Matt Hanson	IM Florida	2:41:57
2021	Gustav Iden	IM Florida	2:34:50*

*Represents a new world record.

According to our estimation, the IM marathon time of a single triathlete performing a 7-h IM would need to be 2 h 25 min and 47 sec, corresponding to a mean velocity of 4.8 m·s^−1^ (17.4 km·h^−1^) or a pace of 3 min and 27 sec·km^−1^. This is about 9 min faster (5.8%) than the fastest marathon time ever recorded in an IM (2 h 34 min and 50 sec) and is much faster than the computed theoretical marathon time. This time is so far off from what history tells us can be achieved that it is very unlikely to be accomplished, even with drafting. Moreover, as this is the last part of the race and time deficits could quickly accumulate if the racing triathlete fatigues prematurely or has a ≪ bad ≫ running day, room for errors is required. However, with external assistance where a time of 3 h and 40 min is tenable on the bike, the racing triathlete would still need to run very fast but complete the marathon in a more ≪ reasonable time ≫ of 2 h 35 min 30 sec (considering a 42 min 32 sec swim time and a total transition time of 2 minutes), which is 40 seconds slower than the fastest marathon time ever recorded in an IM (2 h 34 min and 50 sec).

Among the three main physiological determinants of running performance, which are $\dot{V}$O_2max_, lactate threshold and running economy (Joyner et al., 2011), the latter is likely to play the most important role (Saunders et al., 2004). It is defined as the rate of oxygen consumption or energy expenditure spent per unit of distance (Lacour and Bourdin, 2015; Hoogkamer et al., 2017). Therefore, an improvement in running economy indicates that less energy is expended for running at a given speed. Hence, to meet the optimal running time the racing triathlete should do whatever he can to optimize running economy. Many factors can affect running economy, including body weight, leg architecture, muscle fibers composition (Kyröläinen et al., 2000; Lacour and Bourdin, 2015) and the mechanical and morphological properties of the muscle tendon-units (Arampatzis et al., 2006; Kubo et al., 2010; Albracht, K., and Arampatzis, 2013). Albeit triathletes may exercise a certain control over the first factor, i.e., body weight, they certainly have little influence over the remainder factors which are, for the most part, genetically determined. Moreover, triathletes are generally heavier than elite runners because of the upper body muscles required for swimming which affects their running economy.

Running economy could negatively be impacted after swimming and cycling at a high intensity due to muscle damage (Bessa et al., 2008; Lacour and Bourdin, 2015), neuromuscular fatigue (Lepers et al., 2000), elevated core temperature and possibly dehydration (Hausswirth and Lehenaff, 2001). However, the impact of these factors could be substantially dampened if the racing triathlete drafts other cyclists during the cycling part of the race (Bentley et al., 2002). Indeed, under this circumstance, the rate of energy expenditure during cycling would be significantly less, thereby reducing muscle stress and fatigue, the rate of increase in core temperature and subsequently the rate of sweat loss and dehydration.

Running economy will be expected to slowly deteriorate during the marathon (Hausswirth et al., (1996); Guezennec et al., 1996; Hausswirth and Lehenaff, 2001) due to ongoing muscle damage and muscle glycogen depletion (Assumpção et al., 2013). Indeed, based on Brueckner's (1991) findings, energy expenditure is expected to increase by at least 3% over the course of the marathon. This is due, in part, by a change in the running kinematics, such as a greater forward lean (Hausswirth et al., 1997), an increase in stride time variability (Connick and Li, 2015), a decrease in stride length and an increase in stride frequency (Gottschall and Palmer, 2000; Kyröläinen et al., 2000; Place et al., 2004; Connick and Li, 2015).

Fortunately, there are some tools available to triathletes to minimize the decline in running economy that inevitably occurs during the marathon part of an IM. These are discussed in the following lines.

#### Running Shoes

Running economy can be impacted by the biomechanical advantage provided by shoe technology. Since 1960, when Abebe Bikila ran barefoot to set the world record in 2 h 15 min and 16 sec (Hoogkamer et al., 2017), running shoes have evolved from an ethylene-vinyl acetate (EVA) cushioning to air-cushioned materials. Worobets et al. (2014) observed a 1% improvement in running economy between the Adidas boost midsole and a standard EVA cushioning, which is due to a ≪ superior energy storage/return ≫ in the midsole foam (Hoogkamer et al., 2019). This energy storage/return effect of the midsole foam compensates for the detrimental impact of the slightly higher shoe weight, compared to a more minimalist shoe or to running barefoot (Tung et al., 2014; Hoogkamer et al., 2016).

Furthermore, high-tech running shoes nowadays have a carbon plate midsole, which improves their binding stiffness. The carbon fiber plate (CFP) has a ≪ clever lever effect ≫ on the ankle joint mechanism and it has a ≪ stiffening effect ≫ on the metatarsophalangeal joint (Hoogkamer et al., 2019; Nigg et al., 2020; Cigoja et al., 2021). The carbon fiber plate combined with the superior midsole foam result in an improvement in running economy of between 2.8 (Hunter et al., 2019) and more than 4% (Hoogkamer et al., 2018; Barnes and Kilding, 2019), which translates into an improvement in running performance greater than 2% (Muniz-Pardos et al., 2021). Since the introduction of the carbon fiber plate shoes in 2016, all the women's and men's world record in distance ranging from the 5 km to the marathon have been broken (Bermon et al., 2021; Muniz-Pardos et al., 2021).

#### Drafting

The running energy cost can also be reduced by drafting other runners. Since the running speed is considerably less than the cycling speed, the reduction in energy cost associated with drafting is significantly less with running compared to cycling, but it is still beneficial. Hill (1928) observed that the effect of air resistance when running depended on the air density, the velocity and the projected area of the runner. Pugh (1970) studied the effect of different wind speeds on the running cost. He observed that when the headwind speed increased from 0 km·h^−1^ to 16.2 km·h^−1^and to 66 km·h^−1^, the $\dot{V}$O_2_ of a runner running at 15.9 km·h^−1^ increased from 2.9 L·min^−1^, to 3.1 L·min^−1^ and to 5.0 L·min^−1^, respectively. Pugh (1971) also observed that at a speed of 6 m·s^−1^ (21.6 km·h^−1^), running at a distance of 1 meter behind another runner reduced the air resistance by 80%. Drafting is not only beneficial because it reduces the air resistance but also because it reduces the mental effort required to ≪ set and monitor a challenging pace ≫ (Polidori et al., 2020).

Polidori et al. (2020) analyzed Kenesia Bekele's performance at the 2019 Berlin marathon where he won in a time of 2 h 01 min and 41 sec. Bekele used a cooperative drafting strategy and adopted three different drafting positions behind three pacers during the race. Using computational fluid dynamics, they estimated a reduction in metabolic power between 1.9 and 2.8% due to drafting. Furthermore, Schickhofer and Hanson (2021) calculated that the reduction in energy cost when drafting in the best drafting formation possible is 3.5%, which corresponds to an increase in velocity of 2.3% and an improvement in time of 154 seconds for a marathon. Improving the world record IM marathon time by 2 min and 34 sec would still leave a deficit of 6 min and 30 sec compared to the computed theoretical marathon time of 2 h 25 min and 47 sec, thereby highlighting the importance of the cycling portion of the race.

## Recommendations

In this section, we will provide recommendations regarding the transition, swim, bike and run course configurations, the organization of drafting as well as for the location of the event and the climatic conditions that would favor the success of the event.

### Transition zones, swim, cycling and running courses, and strategies to optimize performance

#### Transition zones

It is necessary to limit the time lost for the transitioning between sports and therefore the transition area needs to be optimally positioned in relation to the swim and cycling finish line. This objective will be reached by limiting the running time from the swim and bike finish line to the transition zone. Intuitively, this can be achieved by having the transition area to be as close as possible to the swim and bike finish. If the ideal running course is close to the swim course then only a single transition area would be needed. Otherwise, a second transition area, distant from the first, may be required if the running course chosen for the *sub 7 project* is removed from the swim course.

#### Swim

The swim needs to be in a calm body of water, because waves reduce the swimming velocity by impacting the swimming efficiency (Kjendlie et al., 2018). Furthermore, swimming in the ocean or in a sea is advantageous because of the added buoyancy due to the salt content in the water (Mclean and Hinrichs, 1998). For instance, the two fastest non-current-assisted swimming times in an IM in 2021 (43 min and 28 sec and 43 min and 53 sec) were achieved at IM Cairns, Australia in a calm ocean, with a wetsuit. Also, as mentioned before, swimming in a wetsuit increases velocity. Since the *sub 7 project* would not be eligible as an official world record, a wetsuit could be worn even if the water temperature is higher than 21.9°C. However, swimming in warm water in a full wetsuit increases the risk of hyperthermia, so it is not recommended.

#### Cycling and Running

The fastest triathlon cycling times have been achieved on moderately flat courses such as at IM Challenge Roth, on highways such as at IM Texas and IM Florida and on race car course such as Formula 1 (F1) and Nascar racetracks (Table 5). These courses are optimal to achieve fast bike splits because they are mostly flat. Hence, less energy is wasted to move a bike uphill against the force of gravity, the quality of the road is good, which minimizes rolling resistance, and they have a small number of turns, with potentially the exception of some F1 courses. Regarding this latter point, turns need to be limited such to minimize the amount of kinetic energy lost by breaking or coasting before a turn. Furthermore, turns can be difficult to navigate in a group.

For the current IM world cycling record achieved during the ZTBR, a cycling ramp, similar to a velodrome ramp, was built to allow the triathletes to make a 180° turn without having to slow down. This technology could be used for the *sub 7 project* if it were to occur on an out-and-back course such as a highway. However, since the *sub 7 project* will most likely occur with the help of pacers it would be important to practice riding the velodrome-like ramp in a group to minimize the risks of crashes during the attempt.

With respect to the run course, it must also be flat and not have any sharp turns, since elevation gain increases the metabolic cost and curves require the runners to generate additional centripetal force on the ground with their legs, which also increases the metabolic cost (Taboga and Kram, 2019; Snyder et al., 2021).

#### Meteorological conditions

A significant amount of body heat is produced (Longman et al., 2021) during exercise due to the conversion of chemical energy into mechanical energy, which is a highly inefficient process. The amount of body heat produced is proportional to the exercise intensity. On the other hand, the capacity of the body to dissipate heat to the environment is inversely proportional to the water pressure in the air surrounding the skin and directly proportional to the temperature gradient between the skin and the environment. It is important to limit the gain in body heat during exercise as it may lead to premature fatigue and impair performance (Cheuvront et al., 2010). Hence, the choice of the location and the time of the year for the *sub 7 project* is crucial; the temperature should neither be too warm nor too cold and the relative humidity should ideally be as low as possible. Solar load also would need to be as low as possible to minimize body heat gain, unless the race is being conducted in relatively cool weather.

Knechtle et al. (2019) analyzed the Boston marathon performances from 1972 to 2018 and observed that for every increase of 1°C in the average temperature, the winners' finishing times increased by 20 seconds and the average finishing time of all the finishers increased by 1 min and 47 sec. It is well documented that the optimal temperature for a marathon performance is between 10 and 12°C (El Helou et al., 2012; El Helou et al., 2007) or between 8 and 15°C (Suping et al., 1992). For instance, the location and the time of day and year for the *INEOS 1:59 challenge* were chosen so that the runners could run at a temperature of about 10°C (INEOS 1:59 Challenge, n.d.).

For running performance, the temperature must be low since the effect of convection is minimal due to the relatively slow speed of the runners. However, when cycling, the cooling effect of the wind is significantly more important. Therefore, a temperature of 10 °C may be too cold while cycling, and the racing triathlete would need to stop at the first transition to add some clothes and energy may be lost due to thermogenesis. Therefore, a delicate balance between the ideal temperature for cycling and running should be taken into account. However, a possible scenario could be to choose a region where the IM would start after noon when the temperature is in the low 20°C or slightly lower and the radiative effect of the sun is high, so that the racing triathlete could begin the marathon at sunset when both the temperature and the solar radiation are declining.

#### Altitude

For every 305 m increase in altitude, there is an ~3% reduction in air density (Levine et al., 2008). Therefore, when cycling at altitude, cyclists encounter less air resistance, which results in an improved performance, unless the physiological detriment associated with the altitude is higher than the improvement associated with the reduction in aerodynamic drag (Capelli and di Prampero, 1995; Bassett, 2000; di Prampero, 2000; Padilla et al., 2000; Hahn and Gore, 2001; Atkinson et al., 2003; Heil, 2005). For instance, both the hour record and the 4000 m cycling records were achieved at the Aguascalientes velodrome in Mexico, which is at an altitude of 1800 m (Delaney, 2021; UCI., 2021).

It would therefore be tempting for organizers to consider having the *sub 7* project at altitude. However, altitude is associated with a decrease in performance for aerobic activities due to a decrease in $\dot{V}$O_2max_ and an increase in the relative intensity at any speed or power output (Garvican-Lewis et al., 2014, 2015; Burtscher et al., 2018). Furthermore, since the effect of air resistance while running a marathon is low, the reduction in air density whilst running at altitude is not beneficial since the physiological impairment is considerable.

#### Time of year and location

As previously mentioned, the bike and run courses must be flat, the swim must be in a calm body of water and ideally in salt water and the temperature and humidity must be low, but manageable on the bike without the addition of clothing. One ideal location would be Bahrain, which is where the half-IM world record was broken. The swim would occur at the Bahrain bay which is a calm body of salt water. The bike and run courses would be flat and have a small number of turns. The ideal time of year would be January; it is the coldest month of the year in Bahrain with average temperature ranging between 14.7 and 20°C, the water temperature is about 19.8°C (Weather-Atlas, n.d), rain is sporadic, solar radiation is significantly lower in January (4.8 kWh/m^2^ day compared to over 6.5 kWh/m^2^ day in the summer months) (Alnaser et al., 2014) and sunset occurs at about 5 PM. Therefore, the IM could begin at 13h00 so to have the run start when both the temperature and solar radiation are lower.

### Organization of drafting

**Figure 3:**
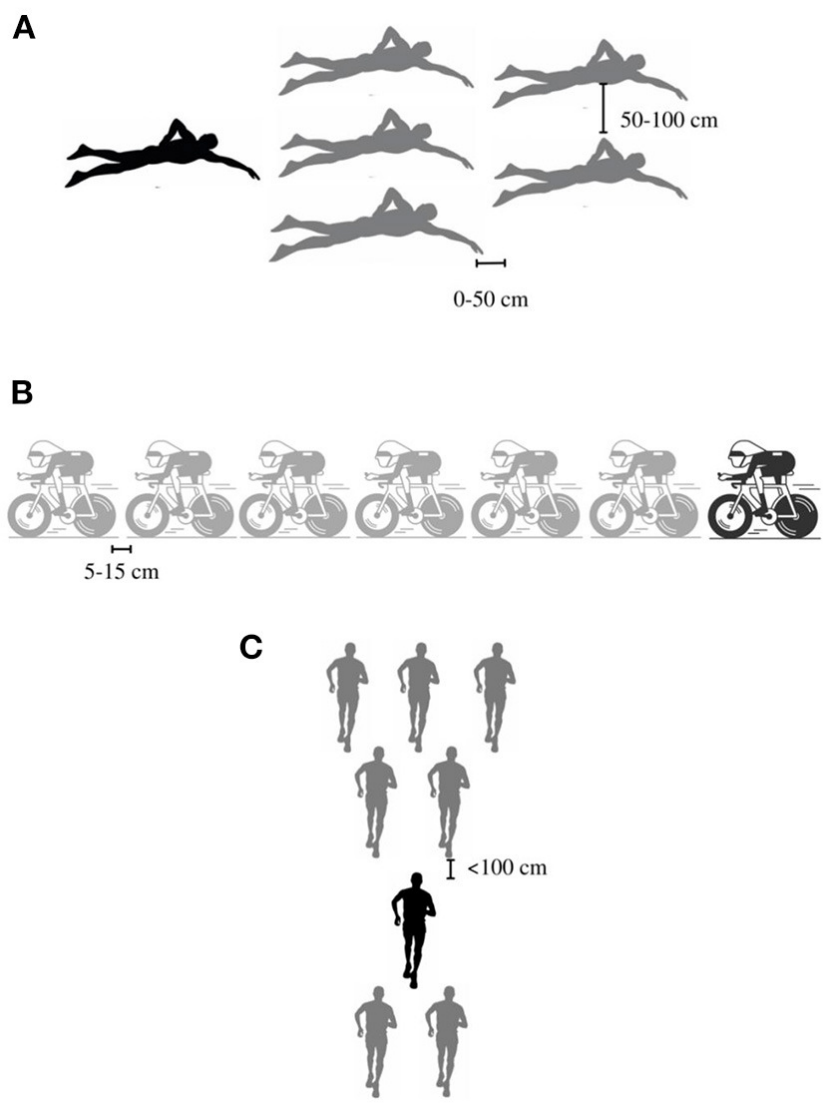
Recommended drafting organizations for **(A)** Swimming, **(B)** Cycling and **(C)** Running. The blackest drawings represent the racing triathlete.

The way drafting will be structured for each of the three sports must be carefully planned and orchestrated for it to provide the best results possible. For the swim, there are two possible positions where a swimmer can be situated to benefit from the draft of other swimmers. One option is to swim between 0 to 50 cm behind the toes of the lead swimmers. The other position, called lateral drafting, requires the swimmer to be located 50 to 100 cm back from the hands of the lead swimmer. However, swimming at the feet is more advantageous (Chatard and Wilson, 2003). In order to maximize the reduction of hydrodynamic drag, the racing triathlete should swim behind five pacers positioned in an arrowhead position (Figure 3A).

For the cycling, based on Blocken et al. (2018)'s study, the optimal situation would be to have 6 lead cyclists in front of the racing triathlete and to try to maintain a space of 5 to 15 cm between each cyclist (Figure 3B). The utilization of radio communication is recommended so that the race organizers and coaches can communicate with the lead cyclists and the racing triathlete. The 6 lead cyclists would need to rotate in order to share the lead and therefore spend short period of times at the front where the aerodynamic drag is the greatest (Shirasaki et al., 2017). They could be replaced by other cyclists at the half point of the 180 km to make sure that the racing triathlete has 6 fresh lead cyclists throughout the entire cycling distance.

Motorcycles could be used to provide on-course food and fluid to the athletes. As a result, instead of having to slow down and get out of his aerodynamic position on his bicycle to grab bottles or gels from an aid station, the racing triathlete would be able to maintain his aerodynamic position and speed, which would allow him to save time and energy. The feeding for the pacers should occur as the lead pacer finishes his pull at the front of the group so that he can coast at the end of the group and receive food and fluids without putting the other cyclists at risk.

For the run, a drafting organization similar to the one used for the sub 2-h marathon attempts is recommended (Figure 3C). For the first sub 2-h marathon attempt, it was decided to have new pacers after every lap of 2.4 km. Kipchoge was running behind another runner who was preceded by 6 runners who were forming an arrowhead. For the second attempt at breaking the 2-h mark, 7 pacers were used, but this time 5 pacers were running in front of Kipchoge in an inverted arrow-head formation and 2 pacers were running behind him (Polidori et al., 2020). For the *sub 7 project*, 7 pacers should be used, and in order to make sure that they can sustain that pace, 7 new pacers would replace them at the half-marathon mark. People riding electric bicycles could ride alongside the runners to provide food, liquids and verbal encouragement.

## Discussion

We determined, based on the trends of the improvement of the IM times over the years, that a sub 7-h IM is unlikely to be achieved without any external assistance. However, this prediction is based solely on trends, so it is not impossible that a sub 7-h IM without any external assistance could be achieved in the future thanks to sophisticated training programs and better monitoring of the recovery process. The arrival of new technologies could also be a game changer. For instance, the use of 3D printing in the development of time trial handlebars with the goal of improving aerodynamism could be extended to create custom, wind-cheating bicycles (Croxton, 2021). Although a taboo topic, it cannot be ruled out that performance-enhancing drugs could be used without being detected, either because of micro-dosing strategies or because the active compound has not been yet been identified by researchers (Joyner et al., 2020). Furthermore, over the past few years, triathletes have begun to compete in IM races at an earlier age which may provide them with more time to improve at that distance while there are still at their peak fitness level.

In this article, courses with a significant negative elevation and with a current-assisted swim were not considered because they fell outside our definition of external assistance. However, a current-assisted swim could help reduce the overall time by as much as 8 min.^7^ Also, a sub 7-h IM could possibly be achieved by swimming in a lake at altitude and then immediately cycling down to a town at low altitude, where the racing athletes would complete their cycling ride. The descent to a lower altitude would allow triathletes to save precious time and energy and enable them to start the run on a fresher body and mind.

The *sub 7 project*, similarly to the *sub2* marathon projects, would require a lot of organization, planning and substantial funding. Furthermore, it would also require a strong commitment in time from the organizers, the pacers and the racing triathlete involved in this event. The triathlete must fully commit to this event, so it should not occur during an Olympic year. Furthermore, he would need to train specifically for this event for many months and to practice the pacing and drafting strategies with the pacers to optimize efficiency and reduce the risks of crashes, especially while cycling. Finally, this achievement would need to be executed, of course, in an ideal location and, most importantly, under perfect meteorological conditions. Therefore, it is recommended to not have the race planned on a fixed day, but rather to plan the race to occur within a spectrum of days ranging from 3 to 5, for instance. Therefore, a careful coordination between race organizers and local authorities and planning of training, diet and hydration for the racing triathlete in the days leading to the race, and within the targeted possible racing days, if need be, would be required.

In conclusion, a sub 7-h IM would be a significant sporting achievement similar to the first sub 4 min mile and the first sub 2-h marathon and physical, psychological and technological boundaries would need to be broken.

## Author contributions

AJD conceived the idea of this article and did research on this topic with the supervision of EDBG. EDBG and AJD performed the analysis of the Ironman^TM^ times. AJD wrote the first draft. EDBG provided guidance on how to present the results, revised the final versions of the manuscript, and the general concepts of this manuscript. All authors discussed the results and contributed equally to the manuscript.

## Conflict of interest

The authors declare that the research was conducted in the absence of any commercial or financial relationships that could be construed as a potential conflict of interest.

## Publisher's note

All claims expressed in this article are solely those of the authors and do not necessarily represent those of their affiliated organizations, or those of the publisher, the editors and the reviewers. Any product that may be evaluated in this article, or claim that may be made by its manufacturer, is not guaranteed or endorsed by the publisher.

## Footnotes

^1^For the purpose of this article, the time of 7 h 27 min and 53 sec will be considered as the current world record, since the swim was not current-assisted.

^2^The 2021 times are not included since the race-course was shorter due to construction work.

^3^The time of 3 h 54 min and 59 sec achieved by American Andrew Starykowicz at IM Florida in 2018 is not considered as the fastest cycling time since the legitimacy of this record is in question because the cycling distance of this triathlon may have been slightly shorter than 180 km.

^4^The fastest unofficial marathon time in an IM is 2 h 34 min and 39 sec and it is held by American Matt Hanson and was achieved at IM Texas. However, the legitimacy of the distance is in question.

^5^Doubling the half-IM time and subtracting an average total transition time of 3 min and 30 sec.

^6^A factor of 5.4% was calculated by Thorsten Raddle (Trirating, 2018) by using a total of 107 data points.

^7^This reduction in time is estimated by comparing the current-assisted swim time to non-current-assisted swim times.
